# Comparative study of serum titanium levels in patients before and after dental implant placement

**DOI:** 10.6026/973206300220175

**Published:** 2026-01-31

**Authors:** Akhilesh Tomar, Akanksha Kashyap, Rakesh Kumar Roy, Shubhashree Ghosh, Vinit Kumar Singh, Harpreet Singh Chhabra

**Affiliations:** 1 Department of Periodontics and Implantology, Vananchal Dental College and Hospital, Garhwa, Jharkhand, India; 2 Department of Periodontics and Implantology, Babu Banarasi Das college of dental sciences, Lucknow, Uttar Pradesh, India; 3 Department of Prosthodontics and Crown & Bridge, Vananchal Dental College and Hospital, Garhwa, Jharkhand, India; 4 Department of Orthodontics and Dentofacial Orthopaedics, Vananchal Dental College and Hospital, Garhwa, Jharkhand, India; 5 Department of Conservative Dentistry and Endodontics, Sardar Patel Post Graduate Institute of Dental And Medical Sciences, Lucknow, Uttar Pradesh, India

**Keywords:** Dental implants, titanium release, systemic absorption, ICP-MS, biocompatibility, tribocorrosion

## Abstract

Titanium (Ti) dental implants are considered the gold standard in edentulous rehabilitation. However, the tribocorrosion and subsequent release of titanium ions into the systemic circulation has become an issue of concern. Hence, this prospective longitudinal study used the Inductively Coupled Plasma Mass Spectrometry (ICP-MS) at baseline, 1-week and 6-months post-operative in order to study serum titanium levels in 60 healthy patients that were undertaking the implant surgery. The baseline serum titanium levels were 1.42 ± 0.55 ng/mL with a statistically significant increase of the mean serum titanium levels to 2.15 + 0.82 ng/mL after one week post- operation and a partial stabilization to 1.94 + 0.71 ng/mL at 6 months. Though, the dental implant placement causes a statistically significant increase in the levels of titanium in the systemic compartments, these levels are far below the accepted toxicological limits.

## Background:

Decades of oral rehabilitation have been based on titanium and its alloys especially Ti-6Al-4V. The success of the material is explained by the fact that it possesses some special properties of the osseointegration, which was first introduced by Brånemark and is based on the stability of the implant-bone interface [1]. Spontaneous formation of a passivation layer, a thin, stable surface film of oxide (TiO2) that covers the underlying metal and prevents corrosion of the underlying metal and allows the cell to attach to it is what dictates the biological inertness of titanium [2]. As a result, the titanium implants are overall considered to be safe with survival rates of more than 95 percent after a decade in a healthy sample. The bio-inert of titanium has however lately been questioned as an increasing amount of evidence indicates that the substance is not completely resistant to breakdown in the extreme electrolytic conditions of the mouth. Implants are exposed to complex physicochemical stress factors in the oral cavity such as changes in pH, bacterial biofilms and mechanical loading, which may also cause tribocorrosion [3]. It is carried out by the synergistic effect of mechanical wear and electrochemical corrosion, which may impair the passivation layer and expose titanium particles or ions into peri-implant tissues [4]. Although it has been reported that local titanium particles in soft tissues build, though with associated conditions, e.g., peri-implantitis, the systemic distribution of these degradation products is controversial. It is established in the orthopedic literature that total hip replacements may significantly increase the level of serum metal ions, to the point of causing systemic toxicity or even hypersensitivity reactions [5]. It is less evident whether dental implants (that have a much smaller surface area and loading mechanics than orthopedic prostheses) raise systemic titanium loads. Recent studies that employed high sensitivity assays have produced divergent findings. A few studies indicate that the friction used during insertion (drilling and screwing) of the implants causes a burst of metal ions although others explain that the levels are insignificant when compared to eating food [6]. Moreover, the kinetics of release of titanium in the early healing stage and the loading phase of the ascertained case of the osseointegration are ill-defined. Therefore, it is of interest to make a comparative analysis of the serum titanium levels in patients before dental implantation and after it with the help of the high-resolution mass spectrometry.

## Materials and Methods:

## Study design and ethical considerations:

This prospective, longitudinal cohort study was conducted at the Department of Periodontology.

## Sample size calculation:

Based on a pilot study indicating a standard deviation of 0.6 ng/mL in serum Ti levels, a sample size of 52 patients was calculated to detect a difference of 0.3 ng/mL with 80% power and a 5% significance level. To account for potential dropouts, 65 patients were initially recruited.

## Inclusion and exclusion criteria:

## Inclusion criteria:

Patients aged 25-65 years requiring 1 to 4 dental implants; adequate bone volume not requiring grafting; systemic health (ASA I or II).

## Exclusion criteria:

History of previous metal implants (orthopedic or dental); presence of amalgam fillings (to rule out other metallosis, though specific to mercury/silver, this was done to ensure a metal-free baseline environment where possible); renal dysfunction (creatinine clearance <90 mL/min); occupational exposure to titanium; intake of titanium-containing supplements or medications (e.g., TiO2 in pills); and smokers.

## Surgical protocol:

All surgeries were performed by a single experienced surgeon to minimize variability. Patients received standard profilaxis. Under local anesthesia, full-thickness flaps were raised. Osteotomies were prepared using a physiodispenser with copious external saline irrigation to prevent overheating. Titanium alloy implants (Ti-6Al-4V, Grade 5) with sandblasted, large-grit, acid-etched (SLA) surfaces were placed. Torque values were recorded. Healing abutments were placed (non-submerged protocol). No bone grafts or membranes were used.

## Blood sampling protocol:

Venous blood samples (5 mL) were collected from the antecubital fossa at three time points:

[1] T0 (Baseline): 1 hour prior to surgery.

[2] T1 (Post-operative): 7 days after surgery (suture removal visit).

[3] T2 (Follow-up): 6 months after surgery (prior to prosthetic loading).

To prevent contamination, samples were drawn using trace-element-free tubes (Royal Blue cap, BD Vacutainer) and stainless-steel needles were avoided; plastic cannulas were used after initial puncture. Samples were allowed to clot for 30 minutes and centrifuged at 3000 rpm for 10 minutes. The serum was aliquoted into metal-free polypropylene cryotubes and stored at -80°C until analysis.

## Analytical procedure (ICP-MS):

Serum titanium concentrations were quantified using Inductively Coupled Plasma Mass Spectrometry (ICP-MS). The serum samples were digested using ultrapure nitric acid and hydrogen peroxide in a microwave digestion system. The solution was diluted with deionized water. Isotope Ti-47 was monitored to reduce polyatomic interferences. The Limit of Detection (LOD) was determined to be 0.05 ng/mL. Calibration curves were generated using standard titanium solutions.

## Statistical analysis:

Data were analyzed using SPSS software (Version 26.0). The Shapiro-Wilk test was used to assess the normality of distribution. Descriptive statistics (Mean ± SD) were calculated. Repeated measures Analysis of Variance (ANOVA) with Bonferroni correction was used to compare Ti levels across T0, T1 and T2. Pearson correlation coefficients were calculated to assess the relationship between the number of implants and serum Ti levels. A p-value of <0.05 was considered statistically significant.

## Results:

Of the 65 recruited patients, 5 were excluded due to failure to attend follow-up or haemolysis of blood samples. The final analysis included 60 patients (32 females, 28 males) with a mean age of 48.2 ± 9.4 years. A total of 110 implants were placed. The distribution of implants was: 25 patients received 1 implant, 20 received 2 implants and 15 received 3 or 4 implants. Table 1 summarizes the baseline characteristics. There were no significant differences in baseline titanium levels regarding age or gender (p > 0.05). The quantitative analysis revealed a statistically significant fluctuation in serum titanium levels over time. At baseline (T0), the mean serum titanium level was 1.42 ± 0.55 ng/mL. One week post-surgery (T1), the mean level rose significantly to 2.15 ± 0.82 ng/mL (p < 0.001). At the 6-month follow-up (T2), levels decreased slightly to 1.94 ± 0.71 ng/mL but remained significantly higher than the baseline (p < 0.05). These results are detailed in Table 2. A subgroup analysis was performed to determine if the quantity of titanium implanted influenced systemic levels. Patients were categorized into Group A (1 implant) and Group B (Multiple implants: 2-4). Group B showed a significantly higher percentage increase in titanium levels at T1 compared to Group A. Pearson correlation analysis demonstrated a moderate positive correlation between the number of implants and the serum Ti concentration at T1 (r = 0.58, p < 0.001) (Table 3).

## Discussion:

This research paper has shown that the surgical insertion of dental implants leads to statistically significant (but numerically insignificant) rise of titanium levels in serum. The findings show that the highest titanium levels occurred after the operation one week later with a continued increase of the levels being detected six months after the baseline. This proves the hypothesis that dental implants are not biologically inert, but they are dynamically interacting with the host by releasing the trace elements. Our baseline value (1.42 ng/mL) agrees with the known reference range of unexposed populations in the literature of the epidemiological literature (in which titanium is generally detected through dietary intake (e.g., TiO2 in food additives, toothpaste and cosmetics)) [7]. But the sudden rise at T1 (2.15 ng/mL) indicates instant release. This is possibly because of the mechanical friction that is created in the course of inserting the implant into the osteotomy site. When the implant surface rubs against the thick cortical bone it may strip away the protective oxide coating and results in the release of titanium particles and ions prior to repassivation [8]. This is actually referred to as the run-in period of wear that has been widely reported in orthopedic literature but is not taken seriously in the field of dentistry [9]. The fact that the levels remained high at 6 months (T2) even though they slightly decreased compared to T1 is an important result. It implies that the initial burst of surgery decreases but a constant amount of release continues to be chronic or that the clearance of the systemic titanium is slow. Titanium is mainly released through urine; however, its half-life in serum can be enlarged in case it is bound to serum protein such as transferrin or albumin [10]. Besides, the conditions of the healing process, which are inflammatory cytokines and oxidative stress, could favor corrosion of the implant surface and sustain elevated serum levels [11].

The correlation of the number of implants and serum levels also supports the origin of the metal. The patients who had more than one implant were found to increase at T1 by 62.7 as compared to a 34.0 increase in single-implant patients. This dose-response correlation is in line with the results of Sarmiento-Gonza *et al.* who observed that the total surface area is a predictor of metal ion release [12]. It disputes the idea that release of dental implant is insignificant, indicating that the systemic load may be significantly greater in the full-arch cases of dental rehabilitation. Although the results of statistical significance, the clinical implications of such studies should be viewed with caution. Our top level of 4.10 ng/mL is several folds lower than the levels of patients with failed metal-on-metal hip prosthesis which may have levels up to 50-100 ng/mL [13]. Toxicological threshold of serum titanium is yet to be developed; however in vitro analysis has indicated that cellular toxicity would be detected at levels much higher (ppm range as opposed to ppb) [14]. Thus, we could not have found proof of acute toxicity. Nonetheless, the possibility of immunological sensitization should not be eliminated. Other scientists maintain that haptens can be as little as trace levels of titanium ions which can induce type IV hypersensitivity reactions in individuals with the susceptibility [15]. This has been found to be involved in unexplained failures of the implants or aseptic loosening, albeit a debatable issue in periodontology [16]. The study has limitations. The follow-ups were shortened to 6 months which was the period of osseointegration and not the functional loading period. The fretting corrosion in the implant-abutment interface may be brought about by the masticatory forces and result in additional release [17]. Also, we failed to examine urine samples, which would have given us the information on renal clearance rates. Further research can be performed on long-term longitudinal follow-ups of 5-10 years and examine the possible relationships between serum Ti levels and biomarkers of inflammation.

## Conclusion:

Although the circulation of titanium increases as more implants are placed, it remains far below established toxicological limits. Therefore, titanium dental implants should not be regarded as hazardous forms of treatment. However, they should not be considered completely bio-inert and the long-term systemic effects of chronic metal exposure especially in patients with multiple implants or metal hypersensitivities warrant observation.

## Figures and Tables

**Table 1:** Demographic and clinical characteristics of the study population

**Variable**	**Value (N=60)**
Age (Mean ± SD)	48.2 ± 9.4 years
Gender (Male/Female)	28 / 32
Total Implants Placed	110
Implants per Patient (Mean)	1.83
Implant Location (Maxilla/Mandible)	62 / 48
Mean Insertion Torque	38.5 ± 5.2 Ncm

**Table 2:** Comparative analysis of mean serum titanium levels (ng/mL) at different time intervals

**Time Interval**	**Mean (ng/mL)**	**SD**	**Range**	**p-value (vs T0)**
T0 (Pre-operative)	1.42	0.55	0.45 - 2.60	-
T1 (1 Week Post-op)	2.15	0.82	0.90 - 4.10	< 0.001*
T2 (6 Months Post-op)	1.94	0.71	0.80 - 3.55	0.012*
Repeated Measures ANOVA;
* indicates statistical significance.

**Table 3:** Serum titanium levels (ng/mL) stratified by number of implants placed

**Group**	**N**	**T0 (Baseline)**	**T1 (1 Week)**	**T2 (6 Months)**	**% Increase (T0 to T1)**
Group A (1 Implant)	25	1.38 ± 0.48	1.85 ± 0.55	1.62 ± 0.50	34.00%
Group B (2-4 Implants)	35	1.45 ± 0.60	2.36 ± 0.91	2.16 ± 0.78	62.70%
p-value (Between groups)		0.621	0.018*	0.004*	
Independent t-test between
groups at each time point
